# Evaluating pretreatment serum CA-125 levels as prognostic biomarkers in endometrial cancer: a comprehensive meta-analysis

**DOI:** 10.3389/fonc.2024.1442814

**Published:** 2024-09-27

**Authors:** Zhong Yu, Yue Sun, Cuishan Guo

**Affiliations:** Department of Obstetrics and Gynecology, Shengjing Hospital of China Medical University, Shenyang, Liaoning, China

**Keywords:** endometrial cancer, CA-125, prognosis, survival, meta – analysis

## Abstract

**Background:**

In recent years, the incidence of endometrial cancer (EC) has been rising. This meta-analysis aims to clarify the prognostic significance of serum CA-125 levels in EC.

**Methods:**

Articles up to March 1, 2024, were systematically searched in EMBASE, Cochrane Library, PubMed, and Web of Science. This analysis pooled hazard ratios (HR) and 95% confidence intervals (CI) from qualifying studies to evaluate the association of CA-125 levels with overall survival (OS), progression-free survival (PFS), disease-free/relapse-free survival (DFS/RFS), and disease-specific survival (DSS).

**Results:**

25 studies involving 7,716 patients were included. The analysis revealed that elevated CA-125 levels correlate with poorer OS (HR = 1.848, 95% CI: 1.571-2.175, p < 0.001). This association persisted across various study regions and sample sizes, and was notably strong in subgroups with a CA-125 cut-off value of less than 35 (HR = 2.07, 95% CI: 1.13-3.80, p = 0.019) and equal to 35 (HR = 2.04, 95% CI: 1.49-2.79, p < 0.001), and among type II pathology patients (HR = 1.72, 95% CI: 1.07-2.77, p = 0.025). Similarly, high CA-125 levels were linked to reduced PFS, particularly in subgroups with a CA-125 cut-off value less than 35 (HR = 1.87, 95% CI: 1.15-3.04, p = 0.012) and equal to 35 (HR = 4.94, 95% CI: 2.56-9.54, p < 0.001), and in endometrioid endometrial cancer patients (HR = 2.28, 95% CI: 1.18-4.40, p = 0.014). Elevated CA-125 levels were also indicative of worse DFS/RFS (HR = 2.17, 95% CI: 1.444-3.262, p < 0.001) and DSS (HR = 2.854; 95% CI: 1.970-4.133, p < 0.001).

**Conclusion:**

Serum CA-125 levels before treatment was highly associated with prognosis of EC patients.

## 1 Introduction

Endometrial cancer (EC) ranks among the top three malignant tumors in the female reproductive system, predominantly affecting perimenopausal and postmenopausal women, with rising global incidence over the past two decades (1). In 2020, there were over 417,000 new cases of EC worldwide, resulting in approximately 97,737 deaths (2). Major risk factors for EC include anthropometric indices, diet, physical activity, medical conditions, hormonal therapy, biochemical markers, gynecological history, and smoking (3–6). The primary treatment for EC, as per guidelines, involves comprehensive staging surgery, complemented by radiotherapy, chemotherapy, hormonal therapy, and targeted therapies (7, 8). Early-stage EC usually offers a favorable outlook with a recurrence rate between 10%-15%, whereas advanced-stage EC, particularly stage IV, has a poor prognosis with a five-year survival rate of only 15% (9).

CA-125, a macromolecular sugar chain antigen, is linked to tumorigenesis, cell proliferation, and metastasis (10–12) in various malignancies (13–15). Despite its sensitivity as a tumor marker, CA-125 levels can be elevated due to various factors such as menstrual periods, pregnancy, inflammation, radiation damage, benign ovarian tumors, and heart disease (16–18). CA-125 level correlates with EC clinicopathological features and predicts lymph node metastasis or extra-uterine spread in advanced cases (19–21). This study aims to elucidate the contentious role of pretreatment serum CA-125 levels in prognosticating EC, thereby contributing to the resolution of existing academic debates (22–24).

## 2 Materials and methods

### 2.1 Search strategy

This Preferred Reporting Items for Systematic Reviews and Meta-Analyses (PRISMA)-compliant meta-analysis (CRD42023443479) (25) searched PubMed, Web of Science, Embase, and Cochrane Library up to March 1, 2024, using keywords related to EC and CA-125. (Endometrial Neoplasm OR Endometrial Carcinoma OR Endometrial Cancer OR Endometrium Cancer OR cancer of the endometrium OR Carcinoma of Endometrium OR Carcinoma of Endometrium OR Cancer of Endometrium OR Endometrium Cancers) AND (CA-125 OR CA 125 OR Carbohydrate antigen 125 OR Cancer antigen 125) AND (prognosis OR prediction)) (search details were showed in the **Supplementary Tables 1**–**4**). Additionally, references from selected articles and grey literature were reviewed to ensure inclusion of all relevant studies. Since the data used in this article were extracted from previous literature, no patient consent or ethical approval was required.

### 2.2 Eligibility criteria

Studies were included if they: (1) diagnosed EC pathologically or clinically; (2) measured pre-treatment serum CA-125 with a specified cut-off; (3) provided hazard ratios (HRs) and 95% confidence intervals (CIs) or sufficient data to calculate them; (4) reported survival outcomes [overall survival (OS), progression-free survival (PFS), disease-free survival (DFS), relapse-free survival (RFS), disease-specific survival (DSS), or cancer-specific survival (CSS)]; (5) were full-text; and (6) were in English. Exclusion criteria were: (1) non-original articles; (2) *in vitro* or animal studies; (3) duplicates; and (4) insufficient data.

### 2.3 Data collection and quality assessment

Data collection was performed by two independent investigators, with disputes resolved by a third investigator. Extracted information included study characteristics, patient demographics, and survival outcomes. HRs were sourced from either multivariate or univariate analyses. In cases where HRs and CIs were not directly provided, they were calculated using Kaplan-Meier survival curves (26). Due to a limited number of studies addressing RFS (only two included), survival data were grouped into OS, PFS, disease-free/relapse-free survival (DFS/RFS), and DSS categories for analysis to enhance statistical robustness. The Newcastle-Ottawa Scale (NOS) (27) evaluated the methodological quality of the studies, assigning scores from 0 to 9 based on criteria such as patient selection, comparability, follow-up, and outcome accuracy. Studies scoring 6 or higher were deemed high-quality.

### 2.4 Statistical analysis

Pooled HRs and 95% CIs evaluated the impact of pretreatment CA-125 on prognosis. Heterogeneity among studies was measured using the Chi-squared test and *I^2^* value, with *I^2^* > 50% indicating significant heterogeneity and prompting a random-effects model (28). A fixed-effects model was used for lower heterogeneity. Subgroup analyses were conducted to identify the sources of heterogeneity, considering variables such as study region, sample size, pathology classifications, CA-125 threshold values, and data origins. The integrity of the results was verified through sensitivity analysis, while Egger’s test investigated publication bias, with adjustments made using the trim-and-fill method where necessary (29). Statistical computations were conducted using STATA 15.0 with HR > 1 indicating poorer survival and significance at 95% CI not intersecting 1 and a p-value less than 0.05 in a two-sided test.

## 3 Results

### 3.1 Study retrieval, selection, and characteristics

**Figure 1:**
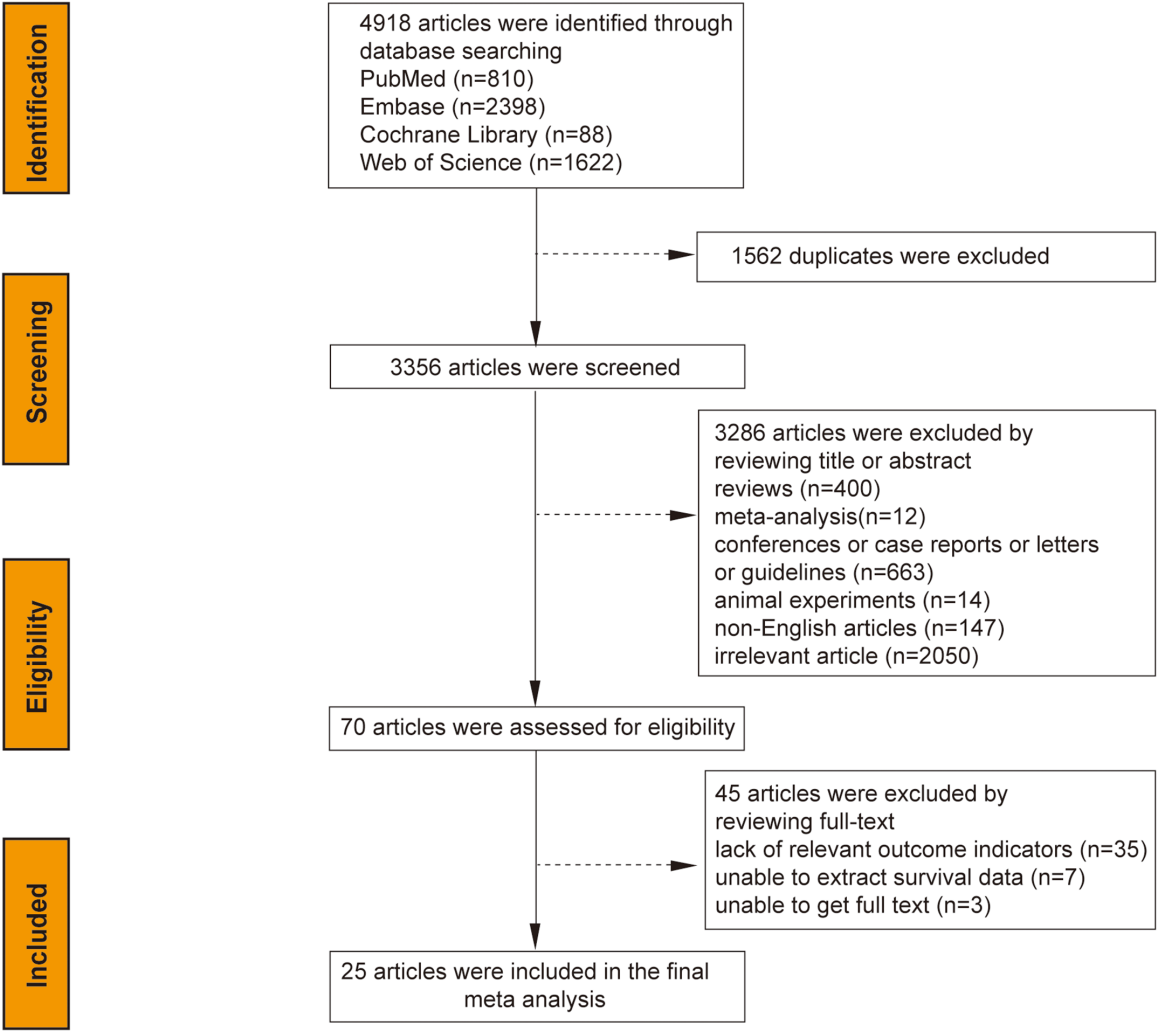
Study selection flow diagram.

The initial search yielded 4,918 articles from the system database; after removing 1,562 duplicates and excluding 3,286 based on title/abstract analysis, 70 articles were fully reviewed. Of these, 45 were excluded for lacking relevant outcome indicators (35 articles), inability to extract survival data (7 articles), or unavailability of the full text (3 articles). Ultimately, 25 studies were selected for meta-analysis, as illustrated in the search flow chart (**Figure 1**). The 25 studies, spanning from 1997 to 2023, encompassed 7,716 patients with sample sizes ranging from 40 to 1,483. Study breakdown included 20 assessing the correlation between CA-125 levels and OS, 10 on DFS/RFS, six on PFS, and three on DSS. CA-125 cut-off values varied from 18 to 70.8 U/mL. Most studies (24) were retrospective, with one prospective study. Geographic distribution included 9 studies from Europe, 13 from Asia, and 3 from the Americas. Statistical analyses employed multivariate methods in 19 studies, univariate in two, and survival curves in 4. Specific cancer types analyzed were endometrioid endometrial cancer (EEC) in 2 studies and Type II EC, including uterine carcinosarcomas (UCSs), uterine papillary serous carcinoma (UPSC), and mixed Type II EC (G3 endometrioid and non-endometrioid cancers) in 5 studies. Seventeen studies covered mixed pathological types of both Type I and Type II EC. All studies achieved NOS scores between 6 and 9, indicating high quality (**Table 1**).

**Table 1:** Characteristics of included studies at baseline.

Author	Year	Country	Study design	Sample size	Age	Treatment	Types of pathology	Lymph node metastasis	CA-125 cut-off value (U/mL)	Data source	Survival outcome	NOS score
Lo, S. S. T. et al	1997	Hongkong	retrospective	97	57.3	not mentioned	Type I and Type II	–	35	Multivariate analysis	OS	6
Sood, A. K. et al	1997	USA	retrospective	210	62.7(27-93)	S	Type I and Type II	15.23%	35	Survival curve	OS	8
Huang, G. S. et al	2007	USA	retrospective	95	65	S alone, S + adjuvant (RT+/- CT)	Uterine carcinosarcomas (UCSs)	–	30	Univariate analysis	OS	7
Olawaiye, A. B. et al	2008	USA	retrospective	41	68(46-87)	S alone, S + adjuvant (RT+/- CT)	Uterine papillary serous carcinoma (UPSC)	39%	35	Multivariate analysis	OS	8
Kim, H. S. et al	2010	Korea	retrospective	413	52(25-83)	S alone, S + adjuvant (RT+/- CT), RT+/- CT	Endometrioid endometrial cancer(EEC)	11.10%	18	Multivariate analysis	OS, PFS	8
Pinar Cilesiz Goksedef, B. et al	2011	Turkey	retrospective	98	60(32-80)	S	Endometrioid endometrial cancer(EEC)	12%	35	Survival curve	OS, PFS	8
Gupta, D. et al	2011	USA	retrospective	52	69.9(55-83 )	S alone, S + adjuvant (RT+/- CT)	Uterine papillary serous carcinoma (UPSC)	20%	30	Multivariate analysis	RFS	8
Chen, Y. L. et al	2011	Taiwan	retrospective	120	54.0(27-79)	S alone, S + adjuvant (RT+/- CT)	Type I and Type II	12.50%	40	Multivariate analysis	RFS	9
Mutz-Dehbalaie, I. et al	2012	Austria	retrospective	183	68(38-89)	S	Type I and Type II	23%	35	Multivariate analysis	OS, DFS	9
Roelofsen, T. et al	2012	Netherlands	retrospective	66	70(51-87)	S alone, S + adjuvant (RT+/- CT), no treatment	Uterine papillary serous carcinoma (UPSC)	50%	45	Multivariate analysis	OS, PFS	8
Chao, A. et al	2013	Taiwan	retrospective	757	52(25-93)	S alone, S + adjuvant (RT+/- CT), RT+/- CT	Type I and Type II	12.28%	35	Multivariate analysis	DSS	8
Li, J. et al	2015	China	retrospective	282	53(21-76)	S	Type I and Type II	4.96%	35	Multivariate analysis	OS, DSS	9
Haruma, T. et al	2015	Japan	retrospective	320	57.5(23-86)	S, preoperative CT+S	Type I and Type II	13.75%	27.95	Multivariate analysis	OS, DFS	9
Harano, K. et al	2016	Japan	retrospective	483	65(35-92)	S alone, S + adjuvant (RT+/- CT)	Uterine carcinosarcomas (UCSs)	–	35	Multivariate analysis	OS, DFS	8
Biler, A. et al	2017	Turkey	retrospective	40	38(21-40)	S alone, S + adjuvant (RT+/- CT)	Type I and Type II	–	35	Multivariate analysis	OS, PFS	7
Kotowicz, B. et al	2017	Poland	retrospective	74	61(42-84)	S alone, S + adjuvant (RT+/- CT)	Type I and Type II	14%	70.8	Survival curve	DFS	8
Abbink, K. et al	2018	Netherlands	retrospective	157	63(56-71)	S alone, S + adjuvant (RT+/- CT)	Type I and Type II	25%	35	Multivariate analysis	OS	9
Reijnen, C. et al	2019	Netherlands	retrospective	333	66(41-89)	S alone, S + adjuvant (RT+/- CT)	Type I and Type II	5.11%	35	Multivariate analysis	DFS, DSS	9
Cymbaluk-Płoska, A. et al	2021	Poland	prospective	349	60.8(36-79)	S	Type I and Type II	17.70%	35	Multivariate analysis	OS, DFS	9
Lin, H. et al	2021	China	retrospective	255	57(50-61)	S alone, S + adjuvant (RT+/- CT)	Type I and Type II	–	35	Multivariate analysis	OS	9
Li, Q. et al	2021	China	retrospective	1038	56(51-61)	S, preoperative CT or RT+S	Type I and Type II	3.66%	34.13	Multivariate analysis	OS, PFS	8
Huang, Y. et al	2021	China	retrospective	246	54.17±8.38	S alone, S + adjuvant (RT+/- CT)	Type I and Type II	7.30%	24.58	Univariate analysis	OS	7
Quan, Q. et al	2021	China	retrospective	191	60(35-89)	S alone, S + adjuvant (RT+/- CT)	Type II (G3 endometrioid and non-endometrioid)	14.66%	46.5	Multivariate analysis	OS, DFS	8
Li, Q. et al	2022	China	retrospective	1483	56(51-61)	S alone, S + adjuvant (RT+/- CT)	Type I and Type II	–	35	Multivariate analysis	OS, PFS	8
Lombaers, M.S. et al	2023	Netherlands	retrospective	333	35-93	S alone, S + adjuvant (RT+/- CT), RT+/- CT	Type I and Type II	29.50%	35	Survival curve	OS, DFS	7

S = surgery; RT = radiotherapy; CT = chemotherapy.

### 3.2 Correlation of pretreatment serum CA-125 level with OS in EC

**Figure 2:**
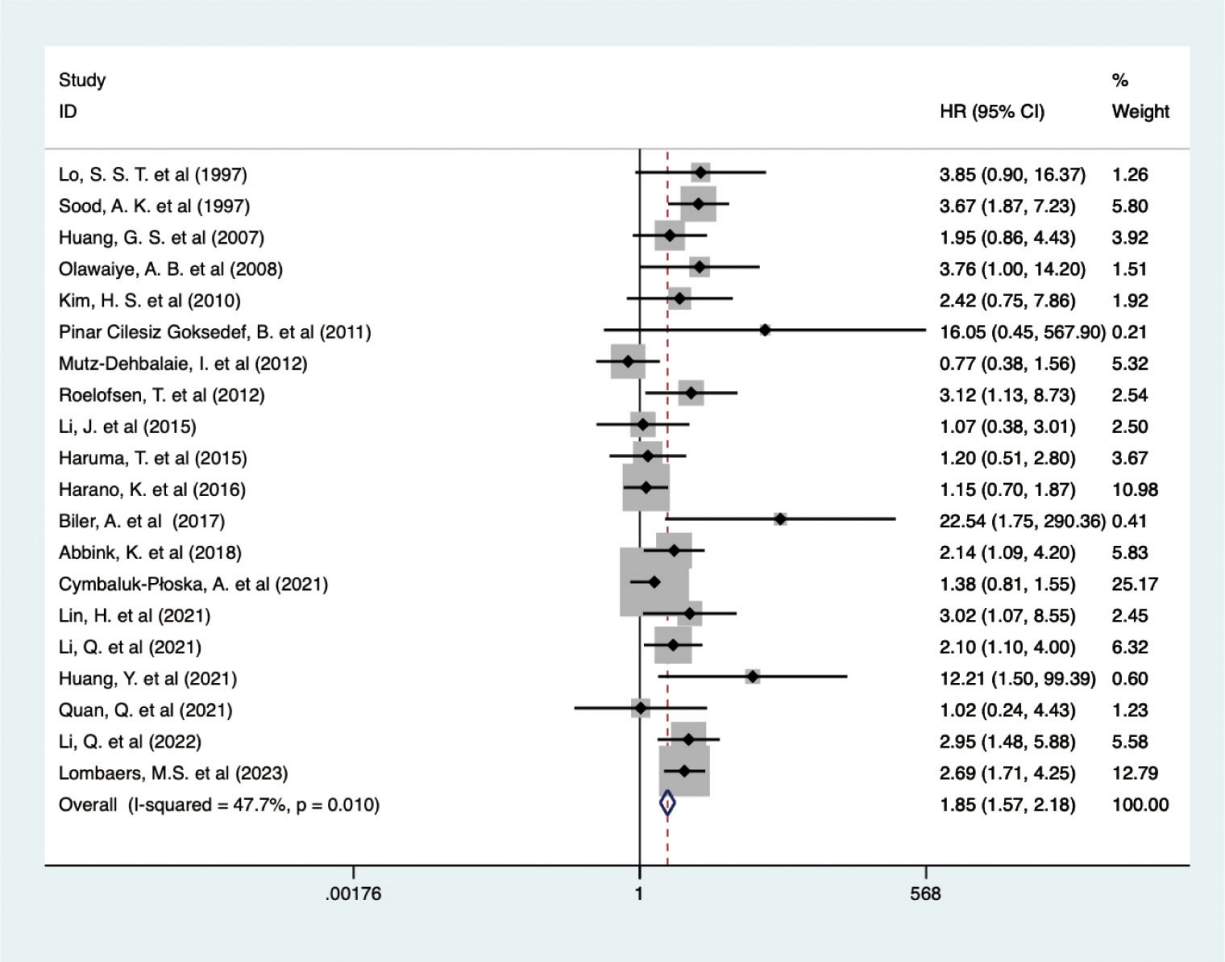
Forest plot of the association between CA-125 and OS in EC.

The analysis of data from 6,380 EC patients across 20 studies revealed that higher pretreatment serum CA-125 levels detrimentally influenced OS (HR = 1.848, 95% CI: 1.571-2.175, p < 0.001) (**Figure 2**) (24, 30–48). A fixed-effects model (I^2^ = 47.7%, p = 0.010), confirmed that higher CA-125 levels adversely affected OS across all study regions and sample sizes. Subgroup analyses revealed a significant negative association between CA-125 and OS for cut-off values ≤35 (HR = 2.07, 95% CI: 1.13-3.80, p = 0.019; HR = 2.04, 95% CI: 1.49-2.79, p < 0.001, respectively) but not >35 (HR = 2.03, 95% CI: 0.70-5.89, p = 0.192). High CA-125 levels were linked with worse OS in type II (HR = 1.72, 95% CI: 1.07-2.77, p = 0.025) and mixed pathology types (HR = 2.10, 95% CI: 1.52-2.91, p < 0.001) but not in EEC (HR = 2.91, 95% CI: 0.95-8.89, p = 0.061). Data source subgroups from multiple analyses (HR = 1.76, 95% CI: 1.34-2.31, p < 0.001) and survival curves (HR = 3.02, 95% CI: 2.07-4.40, p < 0.001) also showed an association between high CA-125 and poorer OS. These detailed findings are summarized in **Table 2**.

**Table 2:** Subgroup analysis of the association between CA-125 and survival outcomes in EC.

Variable		Included studies	Test of association			Effects model	Test of heterogeneity	
			HR	95%CI	*P*		I²	*P*
OS								
Study region	Asia	10	1.87	1.31-2.67	0.001	random	32.10%	0.152
	Europe	7	1.98	1.22-3.22	0.006	random	65.80%	0.008
	America	3	2.95	1.81-4.80	<0.001	random	0.00%	0.469
Sample size	<100	6	3.07	1.83-5.16	<0.001	random	0.00%	0.476
	>100	14	1.84	1.39-2.43	<0.001	random	53.10%	0.01
Cut-off value	<35	4	2.07	1.13-3.80	0.019	random	31.60%	0.223
	35	14	2.04	1.49-2.79	<0.001	random	56.90%	0.004
	>35	2	2.03	0.70-5.89	0.192	random	33.20%	0.221
Types of pathology	mixed	13	2.1	1.52-2.91	<0.001	random	57.50%	0.005
	EEC	2	2.91	0.95-8.89	0.061	random	0.00%	0.324
	Type II	5	1.72	1.07-2.77	0.025	random	27.70%	0.237
Data source	Multivariate analysis	15	1.76	1.34-2.31	<0.001	random	39.40%	0.059
	Univariate analysis	2	3.75	0.67-21.01	0.133	random	60.90%	0.11
	Survival curve	3	3.02	2.07-4.40	<0.001	random	0.00%	0.494
PFS								
Study region	Asia	3	2.47	1.39-4.40	0.002	random	50.70%	0.131
	Europe	3	3.68	0.85-15.92	0.081	random	52.20%	0.123
Sample size	<100	3	3.68	0.85-15.92	0.081	random	52.20%	0.123
	>100	3	2.47	1.39-4.40	0.002	random	50.70%	0.131
Cut-off value	<35	2	1.87	1.15-3.04	0.012	fixed	0.00%	0.611
	35	3	4.94	2.56-9.54	<0.001	fixed	0.00%	0.475
	>35	1	1.56	0.64-3.81	0.329	fixed	–	–
Types of pathology	mixed	3	3.54	1.25-10.02	0.017	random	69.10%	0.039
	EEC	2	2.28	1.18-4.40	0.014	random	0.00%	0.53
	Type II	1	1.56	0.64-3.81	0.329	–	–	–
Data source	Multivariate analysis	5	2.38	1.65-3.42	<0.001	fixed	49.80%	0.093
	Survival curve	1	4.27	0.54-33.63	0.168		–	–
DFS/RFS								
Study region	Asia	4	1.6	0.91-2.82	0.105	random	46.50%	0.132
	Europe	5	2.4	1.34-4.27	0.003	random	92.90%	<0.001
	America	1	2.33	0.80-6.81	0.122	–	–	–
Sample size	<100	2	4.01	2.42-6.65	<0.001	random	27.90%	0.239
	>100	8	1.79	1.33-2.41	<0.001	random	61.60%	0.011
Cut-off value	<35	2	1.51	0.88-2.59	0.137	random	0.00%	0.357
	35	5	1.76	1.26-2.45	0.001	random	70.40%	0.009
	>35	3	4.51	3.46-5.90	<0.001	random	0.00%	0.479
Types of pathology	mixed	7	2.36	1.42-3.93	0.001	random	90.10%	<0.001
	Type II	3	1.43	0.93-2.22	0.107	random	10.40%	0.328
Data source	Multivariate analysis	8	1.81	1.30-2.51	<0.001	random	60.60%	0.013
	Survival curve	2	3.04	1.34-6.91	0.008	random	89.50%	0.002
DSS								
Study region	Asia	2	2.29	1.44-3.64	<0.001	fixed	0.00%	0.709
	Europe	1	4.18	2.27-7.71	<0.001	–	–	–
Data source	Multivariate analysis	2	3.03	1.51-6.10	0.002	random	51.60%	0.151
	Survival curve	1	2.45	1.36-4.41	0.003	–	–	–

### 3.3 Correlation of pretreatment serum CA-125 level with PFS in EC

**Figure 3:**
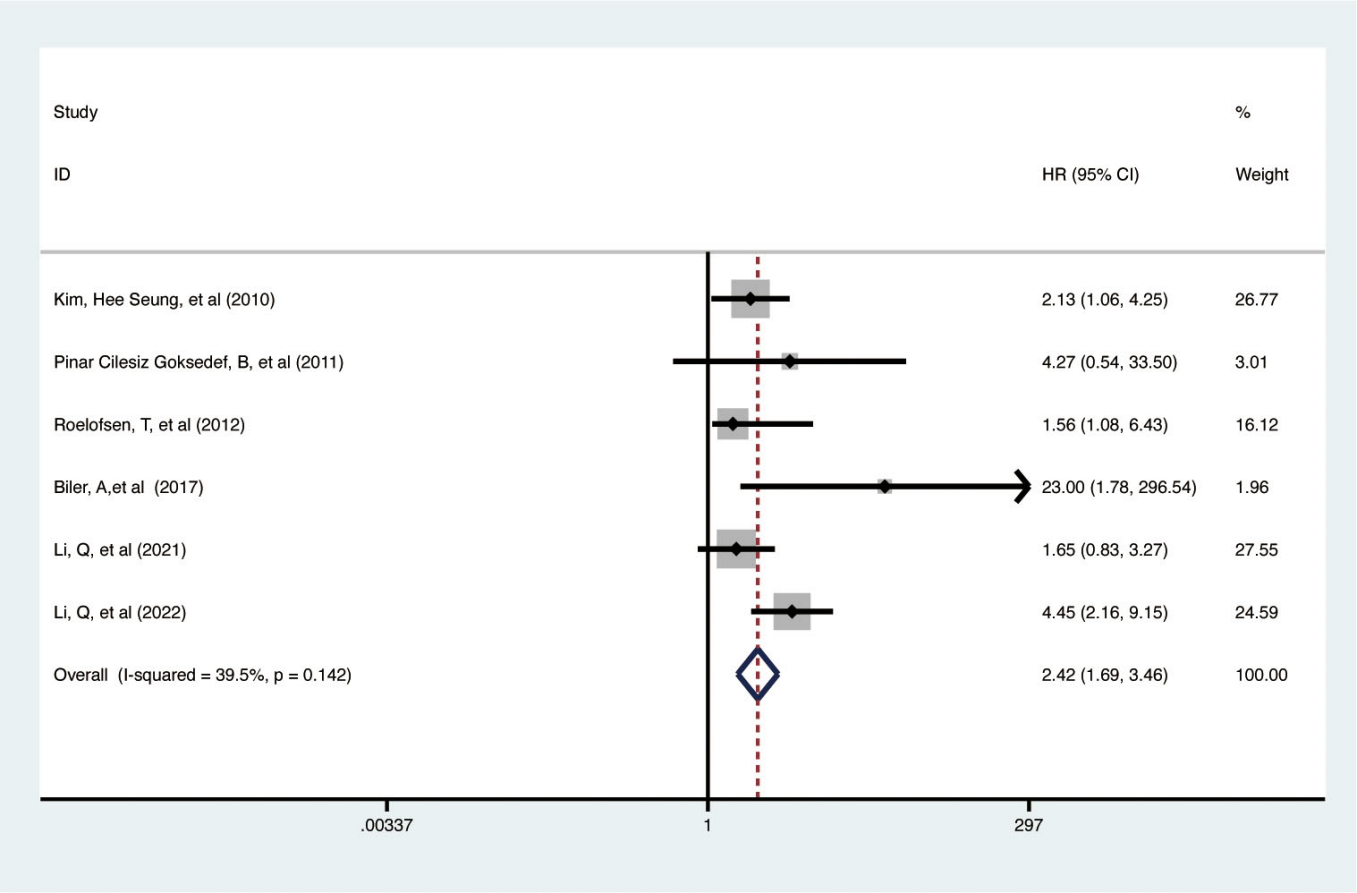
Forest plot of the association between CA-125 and PFS in EC.

Data from 6 studies with 3,138 participants showed that elevated pretreatment serum CA-125 levels were significantly linked to poorer PFS in EC patients (HR = 2.42, 95% CI: 1.692–3.463, p < 0.001) (**Figure 3**) (32, 35, 37, 41, 45, 46). A fixed-effects model was used (I^2^ = 39.5%, p = 0.142). Subgroup analysis revealed that higher CA-125 levels correlated with poorer PFS in Asian EC patients, studies with sample sizes >100 (HR = 2.47, 95% CI: 1.39-4.40, p = 0.002), and cut-off values ≤35 (HR = 1.87, 95% CI: 1.15-3.04, p = 0.012; HR = 4.94, 95% CI: 2.56-9.54, p < 0.001, respectively) but not >35 (HR = 1.56, 95% CI: 0.64-3.81, p = 0.329). Elevated CA-125 was associated with worse PFS in EEC (HR = 2.28, 95% CI: 1.18-4.40, p = 0.014) and mixed pathology types (HR = 3.54, 95% CI: 1.25-10.02, p = 0.017) but not in type II EC (HR = 1.56, 95% CI: 0.64-3.81, p = 0.329). High CA-125 also associated worse PFS in studies with data from multiple analyses (HR = 2.38, 95% CI: 1.65-3.42, p < 0.001). Details of these findings are available in **Table 2**.

### 3.4 Correlation of pretreatment serum CA-125 level with DFS/RFS in EC

**Figure 4:**
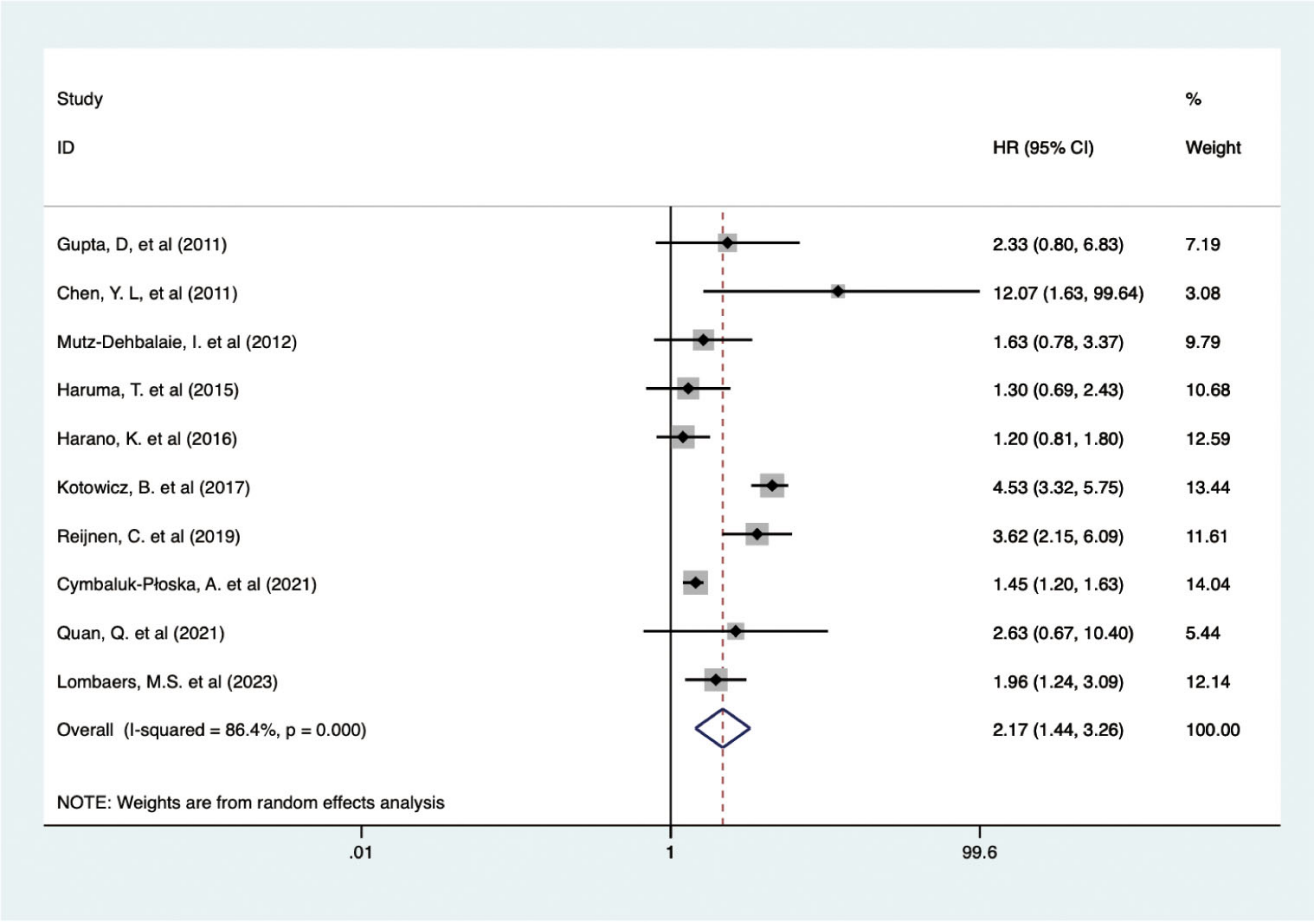
Forest plot of the association between CA-125 and DFS/RFS in EC.

Data from 10 studies with 2,438 patients showed that higher pretreatment serum CA-125 levels were associated with poorer DFS/RFS outcomes in EC (HR = 2.170, 95% CI: 1.444–3.262, p < 0.001) (19, 23, 34, 38, 39, 42, 47–50) (**Figure 4**). A random-effects model was used (I^2^ = 86.4%, p < 0.001). Subgroup analysis demonstrated that elevated CA-125 adversely affected DFS/RFS in European patients (HR = 2.40, 95% CI: 1.34–4.27, p = 0.003), subgroups with CA-125 cutoff values ≥35 (HR = 4.51, 95% CI: 3.46–5.90, p < 0.001; HR = 1.76, 95% CI: 1.26–2.45, p = 0.001, respectively), and patients with mixed pathology types (HR = 2.36, 95% CI: 1.42–3.93, p = 0.001). The negative correlation between high CA-125 levels and poor DFS/RFS was consistent across varying sample sizes and data sources. These results are detailed in **Table 2**.

### 3.5 Correlation of pretreatment serum CA-125 level with DSS in EC

**Figure 5:**
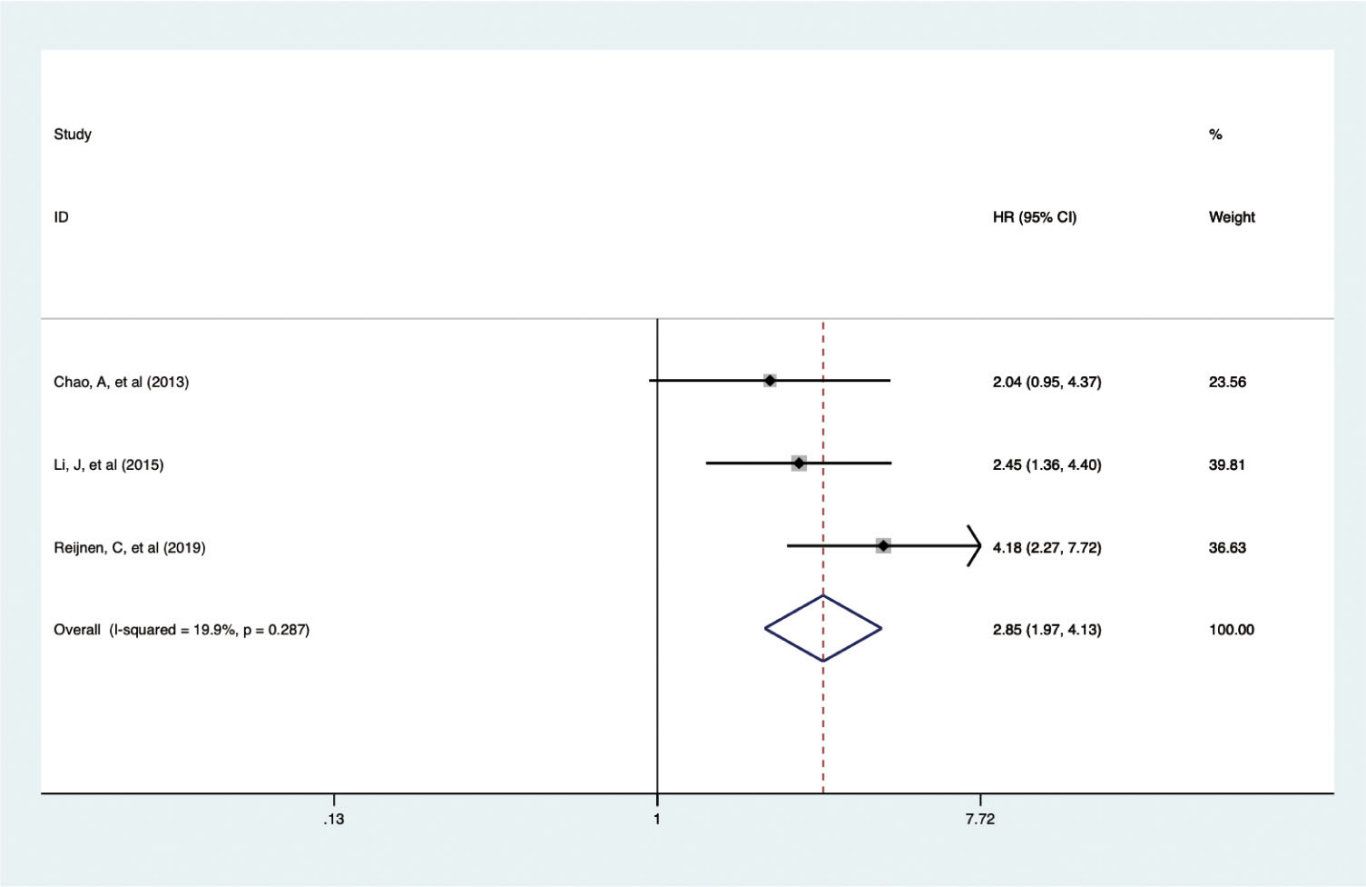
Forest plot of the association between CA-125 and DSS in EC.

In a smaller cohort, three studies with 1,372 patients showed that elevated pretreatment serum CA-125 levels were linked to poorer DSS outcomes in EC (HR = 2.854, 95% CI: 1.970-4.133, p < 0.001) (19, 22, 36) (**Figure 5**). A fixed-effects model was used (I^2^ = 19.9%, p = 0.287). Subgroup analysis indicated that the adverse effects of high CA-125 levels on DSS were consistent across different study regions and data sources, as summarized in **Table 2**.

### 3.6 Sensitivity analysis

**Figure 6:**
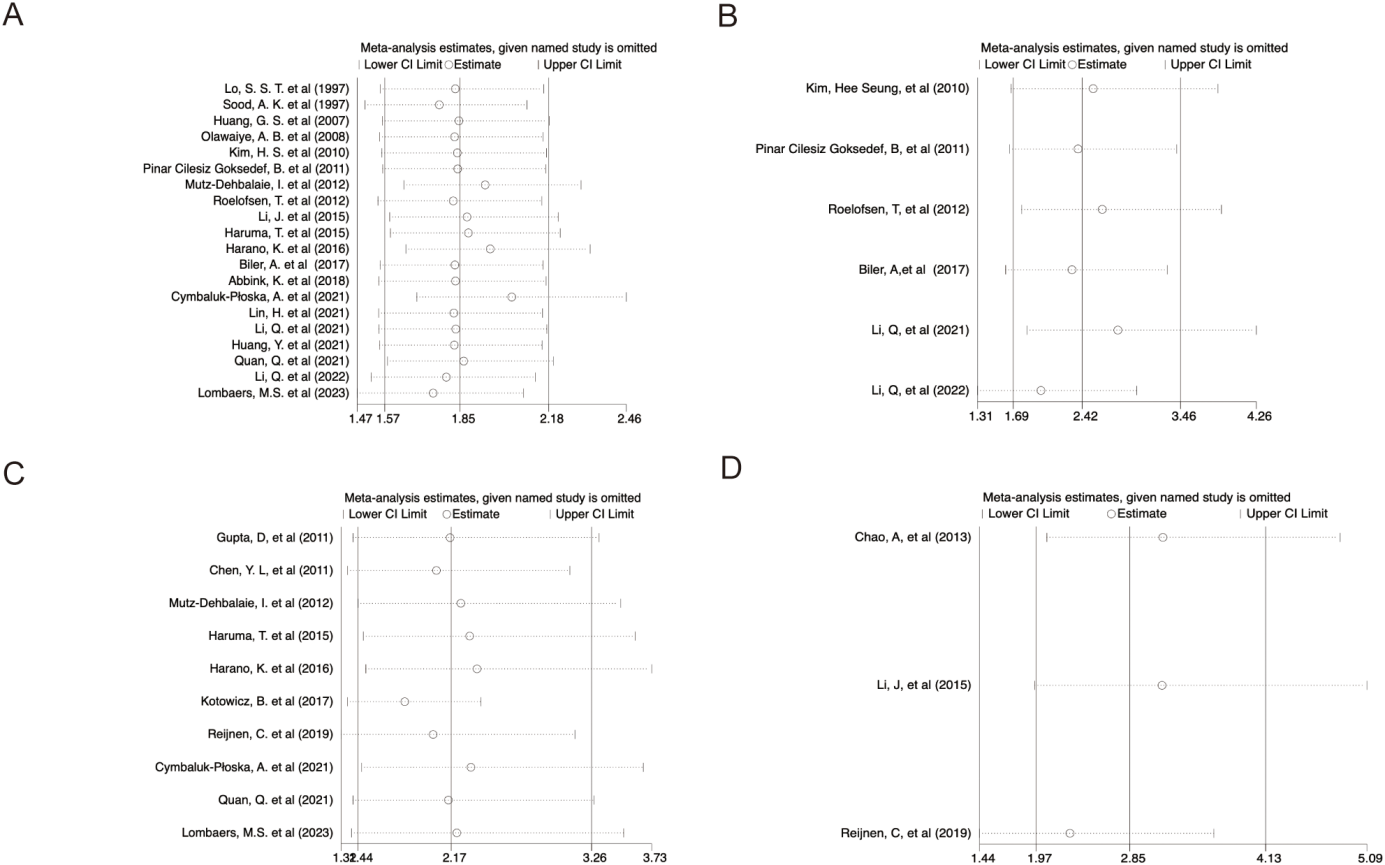
Sensitivity analysis. **(A)** OS; **(B)** PFS; **(C)** DFS/RFS; **(D)** DSS.

Sensitivity analyses using the leave-one-out method demonstrated that excluding any single study from the pool did not significantly alter HRs for survival outcomes, suggesting the meta-analysis results were stable and reliable (**Figures 6A–D**).

### 3.7 Publication bias

Egger’s test detected bias in the OS analysis (p = 0.031) (**Supplementary Figure 1A**). The trim-and-fill method, introducing six hypothetical studies, produced an adjusted HR for OS (HR = 1.762, 95% CI: 1.502-2.067, p < 0.001) (**Supplementary Figure 1B**). The adjusted outcome indicated no significant alteration in the overall effect size, suggesting that the observed bias did not compromise the conclusions. For PFS, DFS/RFS, and DSS, Egger’s tests indicated no significant publication bias (PFS: p = 0.271; DFS/RFS: p = 0.424; DSS: p = 0.670) (**Supplementary Figures 1C–E**).

## 4 Discussion

EC represents a significant gynecologic malignancy within the female reproductive system. Given the rising morbidity and mortality among high-risk and advanced EC patients, identifying prognostic factors is crucial (51). Prior research has highlighted the significance of various surgical and pathological features in prognosticating EC, including FIGO stage, tumor grade, histopathological type, lymph vascular space infiltration, myometrial infiltration, and cervical involvement (52, 53). A preoperative HE4 was associated with tumor’s features and has a good performance in prognosis and monitoring of EC (34, 40, 54). Moreover, molecular characteristics such as DNA mismatch repair deficiency (dMMR), CTNNB1 exon-3 mutation, TP53 mutation, and aberrant p53 expression patterns on IHC have been identified as poor prognostic indicators based on recent TCGA molecular typing (55–60). Additionally, factors like estrogen receptors (ERs), progesterone receptors (PRs), bcl-2, c-erb-B2 (HER2/neu), and proliferation markers (PCNA, Ki-67, MIB-1) are also associated with poor survival in EC (61–63).

CA-125, a well-established biomarker in gynecological malignancies, is crucial for diagnosing, predicting clinical outcomes, and monitoring treatment response in ovarian cancer (OC) (64–66). However, its prognostic value in EC remains contentious. Some studies report that elevated pretreatment serum CA-125 levels correlate with poor EC prognosis (31, 33, 37, 40, 41, 43, 45), while others have produced inconclusive or non-significant findings (22–24, 34, 35, 38, 39, 42, 48). These discrepancies may stem from variations in sample size, patient characteristics, pathological types, and CA-125 cut-off values. To address these inconsistencies, this meta-analysis synthesized data from 25 studies involving 7,716 patients to evaluate the impact of pretreatment serum CA-125 levels on EC survival outcomes, including OS, PFS, DFS/RFS, and DSS.

Elevated pretreatment serum CA-125 levels have been substantially associated with adverse prognostic indicators in EC patients, affirming the marker’s effectiveness in prognostication, similar to findings in OC (67) and other malignancies such as bladder urothelial carcinoma (68), pancreatic ductal adenocarcinoma (69), and renal cell carcinoma (70). This meta-analysis corroborates the pivotal prognostic value of CA-125 in EC, suggesting its potential to enhance prediction of clinical outcomes and guide effective treatment strategies to reduce mortality. Subgroup analyses examining variables such as study region, sample size, cut-off values, pathological types, and data sources revealed no significant differences, reinforcing the consistency of CA-125’s prognostic capacity across diverse clinical settings.

CA-125 is also known to relate closely with clinical pathological characteristics in EC. Higher CA-125 levels are typically linked with extrauterine tumor spread, advanced disease stages (24, 71), and are indicative of lymph node metastasis and greater myometrial invasion depth (72, 73). Furthermore, CA-125 levels vary with different pathological types of EC, being more prevalent in type II EC (48). Subgroup analysis focused on pathological types showed that heightened CA-125 levels significantly correlate with poorer prognosis in both EEC and type II EC, although the studies focusing on a single pathological type were limited.

Previous research often used a CA-125 range of 0–35 IU/mL to determine normal levels. In our meta-analysis, among the 25 studies, 15 studies selected 35 as the cut-off value. The number of studies involving other specific cut-off values is too small to form a subgroup. Therefore, we could only conduct subgroup analysis according to cut-off value equal to 35, greater than 35, and less than 35. Our analysis suggested that when the cut-off value was equal to 35 U/mL, elevated CA125 was associated with all the poor survival outcomes, including OS, PFS, DFS/RFS, DSS. When the cut-off value was greater than 35, elevated CA125 was associated with poor DFS/RFS. When the cut-off value was less than 35, elevated CA125 was associated with poor OS and PFS. According to previous studies, elevated CA-125 was found associated with advanced stages in EC patients, a universal cut-off value of 35 might not accurately reflect disease severity or evaluate prognosis across different EC stages. Because the study objects in all the included articles were patients with EC from stage I to stage IV, future studies should include more detailed stage-specific analyses to refine the prognostic utility of CA-125 in EC.

Serum CA-125, a well-studied tumor biomarker in EC, reflects the expression levels of MUC16, the largest known transmembrane mucin, which is highly expressed in various epithelial cancers (74). The molecular dynamics of CA-125 as a prognostic biomarker are closely linked to the abnormal, high expression of MUC16 in tumor cells, facilitating oncogenesis, proliferation, and metastasis (75–77). Additionally, elevated MUC16 expression is associated with increased chemotherapeutic resistance, metabolic alterations, immune surveillance evasion, and pro-inflammatory signaling (78–80). Mutations in MUC16 also correlate with EC prognosis by enhancing the infiltration of cytotoxic T lymphocytes, which play a critical role in antitumor immunity (81). With numerous clinical trials currently exploring MUC16 as a therapeutic target in OC (82–84), there is growing optimism that targeting MUC16 may similarly improve prognostic outcomes in EC patients.

This meta-analysis identified some intriguing outcomes but also faced several limitations. First, the number of studies analyzing the relationship between CA-125 and various survival outcomes was limited. Second, the predominance of retrospective studies could introduce selection bias. Third, extracting HRs from univariate analyses and survival curves might have resulted in overestimated effects. Additionally, a lack of detailed staging prevented subgroup analyses across different FIGO stages as the studies encompassed all stages collectively. Moreover, the lack of data on menopausal status prevented stratification of analyses. Despite these limitations, the study underscores the potential of CA-125. Cancer’s multifactorial nature often diminishes the accuracy of individual markers (85). A combined approach, integrating various biomarkers with clinicopathological features, is likely to yield more precise and sensitive prognostic assessments (86).

## 5 Conclusions

Generally, this study substantiates the association between elevated CA-125 levels and adverse prognosis in EC, supporting its prospective role as a pivotal molecular biomarker.

## Supplementary material

Supplementary Figure 1 Publication bias assessment of included studies. **(A)** Egger’s test for OS; **(B)** Filled funnel plot using trim and fill method for OS; **(C)** Egger’s test for PFS; **(D)** Egger’s test for DFS/RFS; **(E)** Egger’s test for DSS.

Supplementary Table 1 Search strategy details and results in PubMed.

Supplementary Table 2 Search strategy details and results in Web of Science.

Supplementary Table 3 Search strategy details and results in Embase.

Supplementary Table 4 Search strategy details and results in Cochrane Library.
