# Supplementary figures and images for: Evaluating pretreatment serum CA-125 levels as prognostic biomarkers in endometrial cancer: a comprehensive meta-analysis
Source: Front Oncol. 2024 Sep 27;14:1442814. doi: 10.3389/fonc.2024.1442814 (PMC11466722; doi:10.3389/fonc.2024.1442814)

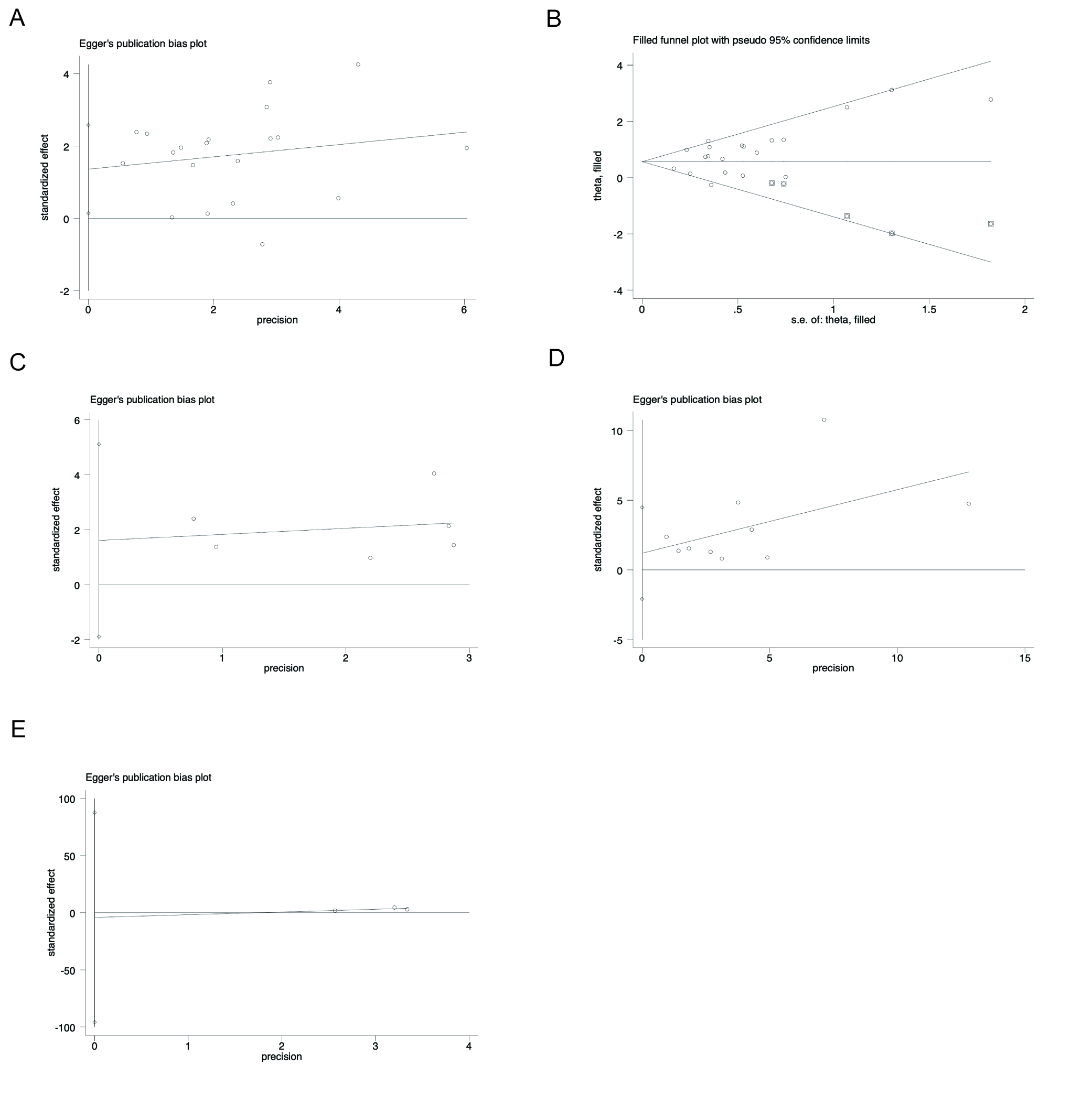

Supplement: Supplementary Figure 1 — Publication bias assessment of included studies. (A) Egger’s test for OS; (B) Filled funnel plot using trim and fill method for OS; (C) Egger’s test for PFS; (D) Egger’s test for DFS/RFS; (E) Egger’s test for DSS. [file Image1.tif]
